# Surgeon Training and Revision Rates After Patellofemoral Arthroplasty

**DOI:** 10.1001/jamanetworkopen.2025.17825

**Published:** 2025-06-27

**Authors:** Louise Eggers Rasmussen, Adam Gorm Hoffmann, Paul Blanche, Frederik Espersen, Tobias Freyberg Justesen, Lasse Enkebølle Rasmussen, Stine Hangaard, Robin Christensen, Anders Odgaard

**Affiliations:** 1 Department of Orthopaedic Surgery, Rigshospitalet, Copenhagen University Hospital, Copenhagen, Denmark; 2 Department of Orthopaedic Surgery, University Hospital of Southern Denmark, Esbjerg, Denmark; 3 Section for Biostatistics and Evidence-Based Research, The Parker Institute, Bispebjerg and Frederiksberg Hospital, Copenhagen, Denmark; 4 Section of Biostatistics, Department of Public Health, University of Copenhagen, Copenhagen, Denmark; 5 Department of Orthopaedic Surgery, Copenhagen University Hospital, Herlev-Gentofte, Copenhagen, Denmark; 6 Now with Department of Orthopaedic Surgery, Hvidovre University Hospital, Hvidovre, Denmark; 7 Now with Department of Surgery, Center for Surgical Science, Zealand University Hospital, Køge, Denmark; 8 Department of Orthopaedic Surgery, Vejle Hospital, Vejle, Denmark; 9 Department of Radiology, Herlev Hospital, Copenhagen, Denmark; 10 Research Unit of Rheumatology, Department of Clinical Research, University of Southern Denmark, Odense University Hospital, Odense, Denmark; 11 Department of Clinical Medicine, University of Copenhagen, Copenhagen, Denmark

## Abstract

**Importance:**

Surgeon training with a specific implant is often not considered in implant registry–based studies, which may lead to unobserved confounding bias. Discrepancies between registry and clinical trial outcomes for patellofemoral arthroplasty (PFA) may originate from differences in surgeon training levels.

**Objective:**

To compare revision rates for knees operated on by knee surgeons specifically trained for PFA and knee surgeons who were not.

**Design, Setting, and Participants:**

In this population-based cohort study, the framework of a target trial was used to compare outcomes for 2 patient groups: patients who underwent PFA performed by knee surgeons who had (trial surgeons) vs who had not (nontrial surgeons) received focused PFA training as part of a randomized clinical trial. All primary PFA procedures from January 1, 2008, through December 31, 2015, were identified using Danish registries and individual hospital notes with 6 years’ follow-up. Data were analyzed from January 24 to March 1, 2024.

**Exposure:**

Focused PFA training.

**Main Outcomes and Measures:**

The primary outcome was 6-year risk of revision. Analyses were conducted according to a prespecified statistical analysis plan, using multiple logistic regression to estimate marginal risk ratios for 6-year risks of revision, reoperation, and mortality, adjusting for potential confounders.

**Results:**

Of 482 eligible knees of patients who had undergone PFA, 274 (57%; 206 female [75%]; mean [SD] age, 61 [13] years) were operated on by trial surgeons, and 208 (43%; 142 female [68%]; mean [SD] age, 57 [12] years) by nontrial surgeons. Trial surgeons operated on knees with higher patellofemoral Kellgren-Lawrence osteoarthritis grade (range 0-4, with 0 indicating no osteoarthritis and 4 indicating severe osteoarthritis) than nontrial surgeons (79% vs 53% with grade 3 to 4) and higher tibiofemoral Kellgren-Lawrence osteoarthritis grades (37% vs 17% with grade 2 to 4). The 6-year revision rate for trial surgeons was 8% (22 of 274 knees) vs 26% (54 of 208 knees) for nontrial surgeons, corresponding to an adjusted relative risk (RR) of 0.35 (95% CI, 0.22-0.56; *P* < .001). The reoperation rate was 12% (33 of 274 knees) for trial surgeons vs 19% (40 of 208 knees) for nontrial surgeons, with an adjusted RR of 0.71 (95% CI, 0.42-1.18; *P* = .19). There was no difference in mortality for trial vs nontrial surgeon groups (18 of 274 knees [7%] vs 12 of 208 knees [6%]; adjusted RR, 1.11 [95% CI, 0.53-2.33; *P* = .79).

**Conclusions and Relevance:**

In this cohort study using a target trial emulation approach to assess the association of surgeon training with PFA outcomes, the cumulative 6-year revision rate for PFA was lower for knees operated on by PFA-trained knee surgeons, suggesting that such surgeon training was associated with a better outcome. This suggests that the level of training may be an important confounder for registry-based comparisons of implant outcome, and that this confounder may even obscure inherent implant outcomes.

## Key Points

**Question:**

Is there an association between focused surgeon training and patellofemoral arthroplasty (PFA) revision rates?

**Findings:**

This cohort study using registry data and a target trial emulation framework involving 482 PFA procedures compared outcomes between surgeons who received focused PFA training as part of a randomized clinical trial (trial surgeons) and those who did not (nontrial surgeons). The cumulative 6-year revision rate was 8% for trial surgeons vs 26% for nontrial surgeons.

**Meaning:**

These findings suggest that implant registry data may be confounded by surgeon training, for instance, when these data are used to compare the revision rates of different knee implant types, with such confounding potentially masking inherent implant properties.

## Teaser

This cohort study uses a target trial emulation framework with registry data to compare revision rates among patients who underwent patellofemoral arthroplasty performed by knee surgeons who were vs were not specifically trained in this procedure.

## Introduction

Isolated patellofemoral osteoarthritis is a degenerative knee condition that causes pain and limits patients’ daily activities. It may occur in as many as 10% of men and 24% of women older than 55 years of age.^1,2^ When conservative treatment fails, arthroplasty may be indicated, and the options are patellofemoral arthroplasty (PFA) and total knee arthroplasty (TKA). Both treatments improve the knee-related quality of life,^3,4^ but there is disagreement on whether one treatment is superior.

Registries are established sources of knowledge regarding the outcomes of surgical treatments.^5^ In the current literature, there is a discrepancy in PFA results reported by registry studies and by randomized clinical trials (RCTs).^6^ While national implant registries consistently demonstrate high early revision rates of PFA,^7^ outcomes reported by RCTs show favorable PFA outcomes compared with TKA,^8,9,10,11^ and even improvements in quality of life in favor of PFA for at least the first 2 postoperative years.^8,9,10^ A possible explanation for the diverging PFA outcomes reported by clinical and registry-based studies may be the level of surgeon training.

Mastering surgical treatments requires knowledge of indications, surgical technique, and complication management. The TKA procedure, the gold standard knee arthroplasty procedure, is far more common than the PFA procedure, and any knee surgeon would have received ample training in it. Because the PFA procedure is performed in far smaller numbers, and training opportunities for it are fewer, the PFA procedure may be at a higher risk of variations of outcome secondary to variations in training. To the best of our knowledge, no implant registry considers surgeon level of expertise with a specific implant, and we speculated that registry observations, in some situations, may primarily reflect the level of surgeon training rather than inherent implant properties. We believe this distinction is important. Therefore, this study aimed to investigate whether the level of surgeon training was associated with the rate of complications, that is, revisions and other reoperations. We used the PFA procedure as an example because of the aforementioned observations that may suggest large variations of outcome.

## Methods

### Study Design

We conducted a retrospective national cohort study of all patients who had undergone PFA in Denmark between January 1, 2008, and December 31, 2015. The study was designed to compare PFA outcomes between 2 groups of patients: those whose knee was operated on by knee surgeons who had received focused PFA training prior to participating in an RCT initiated in 2007 that compared PFA and TKA,^8,10,11^ and those whose knee was operated on by other knee surgeons who neither participated in the RCT nor received focused PFA training. In the current study, these 2 patient groups are referred to as trial surgeon and nontrial surgeon, respectively. The study was registered with ClinicalTrials.gov,^12^ and a statistical analysis plan was prespecified.^13^ Reporting adhered to the Strengthening the Reporting of Observational Studies in Epidemiology (STROBE) reporting guideline statement.^14^ The study was approved by the Danish Data Protection Agency, and permission to access individual patients’ hospital notes was obtained from the National Board of Health and the Capital Region. According to Danish legislation, individual informed consent is not required for register-based studies.

### Target Trial Approach

We used the framework of a target trial emulation for causal inference from observational data.^15^ This approach involves emulating a hypothetical RCT designed to test the falsifiable null hypothesis that patients achieve the same outcome for their knee if they are randomized to either trial surgeons or nontrial surgeons. The framework aims to mitigate the anticipated limitations of causal inference from observational studies, including selection bias and confounding.

The target trial research question was, “Do patients who undergo a PFA procedure under the care of trial surgeons vs nontrial surgeons have the same revision rate 6 years postoperatively?” Patients were eligible for the target trial if they had isolated patellofemoral osteoarthritis and if nonoperative treatment had been unsuccessful. Further eligibility criteria were being 18 years of age or older, having Danish citizenship, being able to consent to participate in an RCT, and having received PFA. Exclusion criteria were severe neurological conditions or disseminated malignant disease. Patients treated conservatively were not of interest to the target trial and were excluded. The primary outcome was the revision rate 6 years after the PFA procedure, and secondary outcomes were the rates of other reoperations and mortality. The knee that underwent PFA was considered the target of the allocation with exposure to either a trial surgeon (intervention) or nontrial surgeon (control). The randomization took place after referral to a participating orthopedic unit where patients were allocated to the care and management of either a trial surgeon or a nontrial surgeon. Time zero of the target trial was the day of surgery, and the preoperative period (eg, for preassessment, imaging) did not count. The main analysis of the trial corresponded to a survivors’ analysis in the terminology of Colantuoni et al,^16^ in which survival indicated that patients survived the preoperative assessment and were offered surgery. Thus, patients from the current study’s cohort were eligible for the target trial emulation if their knee had received PFA within the accrual period, the date of the index PFA was considered time zero, and the patients were followed up for 6 years postoperatively.

### Details of the Exposure Group

All surgeons in the current study were knee specialists. The primary difference surrounding knees operated on by trial surgeons and nontrial surgeons was the surgeons’ level of specific PFA training. Knees operated on by trial surgeons were defined by the surgeon having participated in an RCT that aimed to compare PFA and TKA. The design of the RCT was previously reported to ClinicalTrials.gov,^17^ and patients enrolled in this RCT were operated on from June 2007 to October 2014.^11^ Briefly, before taking part in the RCT, all participating knee surgeons received focused PFA training on September 28, 2007, during an all-day symposium detailing the entirety of the PFA procedure preoperatively, perioperatively, and postoperatively. The Avon implant (Stryker Orthopaedics) was used in all 50 PFA procedures of the trial. Nontrial surgeons were knee specialists who presumably had not received specialist PFA training.

### Data Sources and Data Elements

The main data sources were health administrative registries and hospital notes of individual patients. All PFA operations performed during the accrual period were identified through the Danish Knee Arthroplasty Register (DKR)^18^ and the Danish National Patient Register (DNPR).^19^ Data were extracted from both registries (DKR and DNPR) to obtain the most complete cohort, and knees were included if they were recorded with a primary, cemented PFA procedure (KNGB13). All subsequent revisions (KNGC*), other surgical knee procedures (KNG* except KNGC*), and femoral amputations (KNFQ19) were recorded. All patients’ hospital notes were reviewed manually to collect preoperative, perioperative, and postoperative details, including all available radiographs and surgeon affiliation (trial vs nontrial). In some hospitals, the surgical staff consisted of both trial and nontrial surgeons. Basic personal information was obtained from the Central Personal Register.^20^ Six-year postoperative information was available for all patients without any censoring events. A revision was defined as a procedure in which either 1 or more components were replaced, a component was added, or a component was removed. A reoperation was defined as all other types of surgical procedures on the knee (a revision was not considered a reoperation).

### Statistical Analysis

The primary end point was the occurrence of revision within 6 years after the procedure. Secondary end points were the occurrence of a reoperation (other than revisions) and death within 6 years after the procedure. For the main analyses, we used multiple logistic regression followed by standardization (g-computation)^21,22^ to adjust for confounding and to estimate the marginal risks of revision, reoperation, and mortality within 6 years, for each arm. The risks in the trial surgeon and nontrial surgeon knee groups were compared via risk ratios. Generalized estimating equations (with independence working correlation) were used to fit the model. Robust standard errors were computed via nonparametric bootstrapping by resampling the patients, and not the individual knees, to account for a possible correlation between 2 knees of the same patient. The main analyses were adjusted for 7 potential preoperative confounders: age group (<50, 50-70, >70 years); sex; history of either patella dislocation, knee trauma, or knee dysplasia; duration of symptoms (<5 years vs ≥5 years); any use of analgesics; previous surgery; and year of surgery. We also performed supplementary analyses adjusted for 1 more covariate: either body mass index (BMI), tibiofemoral Kellgren-Lawrence (TF-KL) grade (0-1 vs 2-4), or patellofemoral KL (PF-KL) grade (0-2 vs 3-4) (KL grade range, 0 to 4, with 0 indicating no osteoarthritis and 4 indicating severe osteoarthritis). These variables were neither part of the main analysis nor adjusted for simultaneously in the model because of the substantial proportions of missing data for each of them (Table 1). The choice of the adjustment sets resulted from discussions, prior to running any analyses, between 4 of the trial surgeon knee specialists (including A.O.)^11^ and 2 statisticians (P.B. and R.C.). Complete case analyses were performed in case of missing baseline covariates. For sensitivity analysis, we used propensity score weighting.^21,22^ Propensity scores were also computed via multiple logistic regression, using the same adjustment sets. No interaction term was used in any model. For completeness, crude unadjusted analyses were also performed. Further details can be found in the prespecified statistical analysis.^13^

**Table 1.** Baseline Characteristics of All Included Participant Knees, Stratified by Exposure Group

Characteristic	Participant knees, No. (%)^a^	
	Operated on by trial surgeons (n = 274)	Operated on by nontrial surgeons (n = 208)
Age, mean (SD), y	61 (13)	57 (12)
Sex		
Female	206 (75)	142 (68)
Male	68 (25)	66 (32)
Weight, mean (SD), kg	80 (17)	81 (16)
Not recorded	96 (35)	88 (42)
BMI, mean (SD)	28.0 (5.0)	27.8 (4.6)
Not recorded	97 (35)	101 (48)
Primary diagnosis		
PF-OA (dysplasia or idiopathic)	271 (99)	200 (96)
PF-OA (posttraumatic OA)	2 (1)	4 (2)
Traumatic lesion	1 (<1)	4 (2)
Primary symptom		
Pain	240 (90)	184 (91)
Pain and instability	26 (10)	19 (9)
Not recorded	8 (3)	5 (2)
History of patella dislocation		
Yes	46 (17)	22 (11)
No or not recorded	228 (83)	186 (89)
History of knee trauma		
Yes	29 (11)	37 (18)
No or not recorded	245 (89)	171 (82)
Knee dysplasia		
Yes	21 (8)	8 (4)
No or not record	253 (92)	200 (96)
Symptom duration		
<5 Years or not recorded	166 (61)	115 (55)
≥5 Years	108 (39)	93 (45)
Analgesics		
Yes	175 (64)	125 (60)
No or not recorded	99 (36)	83 (40)
NSAID or paracetamol	151 (55)	108 (52)
Opioids	24 (9)	11 (5)
Tramadol or codeine	43 (16)	39 (19)
Gabapentin	2 (2)	3 (2)
Other analgesics	15 (6)	17 (8)
Previous surgery		
Yes	111 (41)	114 (55)
No or not recorded	163 (59)	94 (45)
Previous conservative treatment		
Yes	137 (50)	95 (46)
No or not recorded	137 (50)	113 (54)
Right knee	156 (57)	108 (52)
Range of motion		
<120 Degrees	46 (18)	42 (26)
≥120 Degrees or to soft tissue	214 (82)	120 (74)
Not recorded	14 (5)	46 (22)
Varus malalignment		
Yes	1 (1)	0
Not recorded	199 (73)	161 (77)
Valgus malalignment		
Yes	8 (11)	7 (15)
Not recorded	199 (73)	161 (77)
Effusion		
Yes	40 (32)	29 (43)
Not recorded	150 (55)	141 (68)
Positive patellar apprehension test		
Yes	8 (36)	23 (85)
Not recorded	252 (92)	181 (87)
Positive Clarke test		
Yes	27 (90)	0
Not recorded	244 (89)	208 (100)
Retropatellar crepitus		
Yes	175 (94)	131 (94)
Not recorded	87	69 (33)
J-sign		
Yes	12 (32)	1 (100)
Not recorded	237 (86)	207 (100)
AP instability		
Yes	2 (1)	4 (3.2)
Not recorded	97 (35)	82 (39)
ML instability		
Yes	2 (1)	1 (1)
Not recorded	93 (34)	78 (38)
Hip pathology		
Yes	15 (8)	6 (10)
Not recorded	95 (35)	145 (70)
Tibiofemoral Kellgren-Lawrence grade^b^		
0	14 (6)	23 (15)
1	126 (57)	107 (68)
2-4	82 (37)	27 (17)
Missing	52 (19)	51 (25)
Patellofemoral Kellgren-Lawrence grade^b^		
0-2	43 (21)	56 (47)
3-4	161 (79)	64 (53)
Missing	70 (26)	88 (42)

Abbreviations: AP, anterior-posterior; BMI, body mass index (calculated as weight in kilograms divided by height in meters squared); ML, mediolateral; NSAID, nonsteroidal anti-inflammatory drug; PF-OA, patellofemoral osteoarthritis.

^a^ Percentages are among nonmissing values.

^b^ Grade range is 0, indicating no osteoarthritis, to 4, indicating severe osteoarthritis.

Additional sensitivity analyses were performed in response to editorial feedback. One analysis consisted of additionally adjusting for the surgeons’ PFA experience, which could be an important confounder. We defined a surgeon’s level of experience for each procedure as the ordinal number of the procedure. At a surgeon’s first PFA procedure, the level of experience was set to 1 and increased with each subsequent procedure, allowing for quantification of experience over time. This variable was adjusted for in the model using linear splines with knots at 5 and 10, to avoid relying on strong modeling assumptions. Another analysis was performed to account for a potentially non-negligible correlation between the outcomes of 2 surgical procedures performed by the same surgeon. Here bootstrapping was performed by 2-step resampling: we first resampled the surgeons and then the patients of each surgeon, both with replacement (eMethods in Supplement 1).

Data were analyzed from January 24 to March 1, 2024, using R, version 4.3.2 (R Project for Statistical Computing). A 2-sided *P* < .05 was considered statistically significant.

## Results

**Figure 1.**
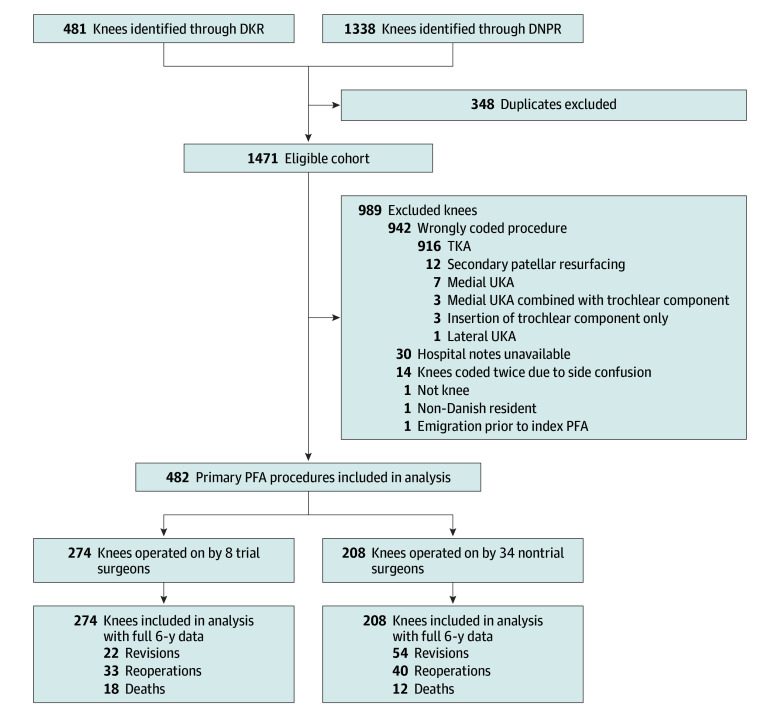
Illustration of Knee Flow From Enrollment to Full 6-Year Outcome Assessment DKR indicates Danish Knee Arthroplasty Register; DNPR, Danish National Patient Register; PFA, patellofemoral arthroplasty; TKA, total knee arthroplasty; UKA, unicompartmental knee arthroplasty.

A total of 482 primary PFA procedures were included in the analysis, of which 274 knees (57%; 206 female [75%] and 68 male [25%]; mean [SD] age, 61 [13] years) were operated on by trial surgeons and 208 knees (43%; 142 female [68%] and 66 male [32%]; mean [SD] age, 57 [12] years) by nontrial surgeons. Overall, 481 potential PFA procedures were identified through the DKR and 1338 through the DNPR during the accrual period. After removing duplicates, the eligible cohort included 1471 knees. Of these knees, 989 were excluded, mainly due to wrongly coded procedures (Figure 1). In total, 410 patients contributed a single knee, and 36 patients contributed both knees. Preoperative radiographs with an anteroposterior view were available for 379 knees (79%), and radiographs with a skyline view were available for 324 knees (67%). Complete 6-year follow-up data were available for all included knees.

The trial surgeon group included 8 surgeons, and the nontrial surgeon group included 34 surgeons. The median (range) numbers of PFA procedures were 28 (11-83) for trial surgeons and 4 (1-33) for nontrial surgeons within the study period. Additionally, trial surgeons performed a total of 44 PFA procedures in the RCT during the study’s accrual period and 288 procedures outside the RCT. At the time of operation, the median (IQR) level of experience value of the operating surgeon was 22 (9-39) in the trial surgeon group compared with 5 (2-9) for the nontrial surgeon group. Overall, the knees were relatively similar across the 2 surgeon groups. Knees operated on by trial surgeons had a mean [SD] age of 61 (13) years) vs 57 (12) years for those operated on by nontrial surgeons. Trial surgeons were more likely to operate on knees with a higher degree of radiographic degeneration as expressed by the TF-KL and PF-KL grades. Among the knees operated on by trial surgeons, 82 of 222 (37%) had a TF-KL grade of 2 to 4, and 161 of 204 (79%) had a PF-KL grade of 3 to 4. In comparison, among knees operated on by nontrial surgeons, 27 of 157 (17%) had a TF-KL grade of 2 to 4, and 64 of 120 (53%) had a PF-KL grade of 3 to 4. Baseline characteristics of the included knees, stratified by exposure group, are presented in Table 1. Trial surgeons mainly used the Avon implant (263 of 274 knees [96%]). This was the implant used in the RCT, and for familiarity reasons, the trial surgeons appeared to also use this implant outside the trial. Conversely, nontrial surgeons used 9 different implants, with the Avon implant being the most used (47 of 208 knees [23%]) and the Hemicap implant (Arthrosurface) the least used (4%). The surgical data of all the included knees are presented in eTable 1 in Supplement 1.

**Figure 2.**
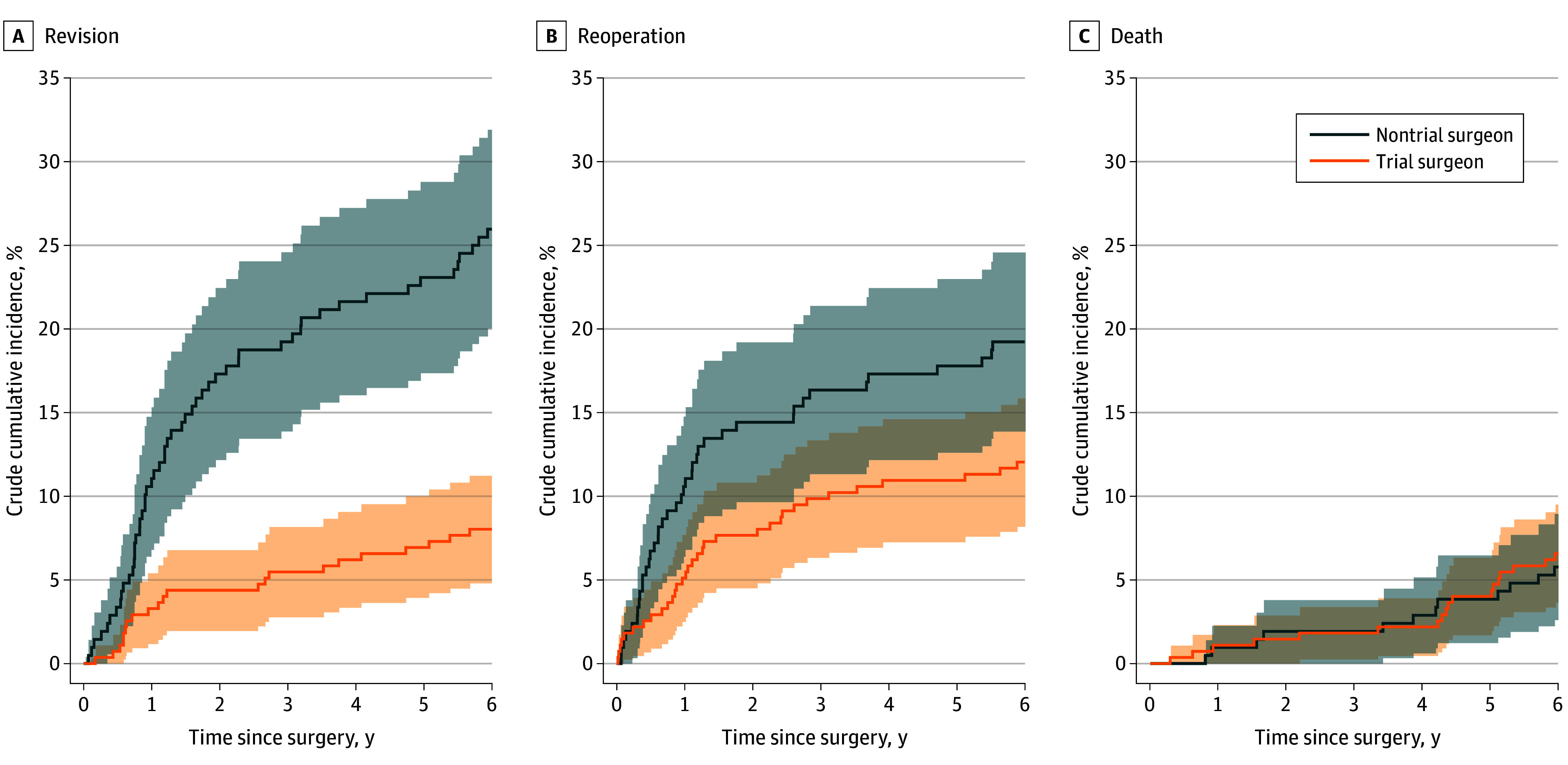
Crude (Unadjusted) Cumulative Incidence of Revision, Reoperation Other Than Revision, and Mortality for Knees Operated on by Trial and Nontrial Surgeons

We observed an 8% revision rate (22 of 274) within 6 years of the index procedure for trial surgeon PFA procedures compared with 26% (54 of 208) for nontrial surgeon PFA procedures (Figure 2). This finding corresponded to a crude relative risk (RR) of revision of 0.31 (95% CI, 0.19-0.49; *P* < .001) for trial surgeon relative to nontrial surgeon PFA procedures (Table 2). After adjusting for potential confounders, the main analysis found an RR of 0.35 (95% CI, 0.22-0.56; *P* < .001) (Table 2). Among trial surgeon PFA procedures, we observed a 12% reoperation rate (33 of 274) within 6 years of the index procedure, while knees operated on by nontrial surgeons had a 19% reoperation rate (40 of 208). The crude RR of reoperation was 0.63 (95% CI, 0.40-0.97; *P* = .04); however, after adjusting for potential confounders, we did not find a difference in the RR (0.71 [95% CI, 0.42-1.18]; *P* = .19) (Table 2). Mortality within 6 years was comparable, 7% (18 of 274) and 6% (12 of 208) for trial and nontrial surgeon PFA procedures, respectively, corresponding to a crude mortality RR of 1.14 (95% CI, 0.52-2.47; *P* = .74) (Table 2). The corresponding adjusted RR of mortality was 1.11 (95% CI, 0.53-2.33; *P* = .79).

**Table 2.** Crude and Adjusted Risk Ratios for the 6-Year Cumulative Revisions, Reoperations Other Than Revision, and Mortality for the Trial Surgeon Group and Nontrial Surgeon Group

Outcome	Knees operated on, No. (%)		Relative risk (95% CI)	
	By trial surgeons (n = 274)	By nontrial surgeons (n = 208)	Crude	Adjusted
Revision	22 (8)	54 (26)	0.31 (0.19-0.49)	0.35 (0.22-0.56)
Reoperation	33 (12)	40 (19)	0.63 (0.40-0.97)	0.71 (0.42-1.18)
Mortality	18 (7)	12 (6)	1.14 (0.52-2.47)	1.11 (0.53-2.33)

In sensitivity analyses, the risk of revision remained similar after additionally adjusting for BMI, TF-KL, PF-KL, or level of surgeon PFA experience, including the propensity score analysis (eTable 2 in Supplement 1). The results were similar when additionally adjusting for TF-KL and surgeon experience, but the relative risks were attenuated when additionally adjusting for either BMI or PF-KL (eFigure 1 in Supplement 1). Although BMI was similar for both patient groups, the high number of missing data points for BMI resulted in a larger statistical uncertainty. The sensitivity analysis that accounted for a potentially non-negligible correlation between the outcomes of 2 surgical procedures performed by the same surgeon also led to similar results for the RR of revision (95% CI, 0.18-0.70 vs 95% CI, 0.22-0.56 for the main analysis). The RRs of reoperation and mortality were similar when additionally adjusting for BMI, PF-KL, TF-KL, and surgeon experience (eFigure 2 and eFigure 3 in Supplement 1).

## Discussion

In this cohort study using an emulation of a target trial framework with regression standardization and propensity score weighting, we compared the outcomes of patients who underwent PFA and whose knees were operated on by 2 groups of surgeons based on registry data merged with data from hospital records. After adjusting for potential confounders, knees operated on by nontrial surgeons had a 3-fold higher 6-year risk of revision compared with those operated on by trial surgeons.

No difference was observed in the 6-year risk of reoperation or mortality between the 2 groups. However, the wide confidence intervals for reoperation risk do not rule out the possibility of a non-negligible association. Trial surgeons predominantly used the Avon implant, whereas nontrial surgeons used a variety of implants. To our knowledge, there is no evidence of superiority of one PFA implant type over others, and it seems unlikely that different implant types used by trial and nontrial surgeons intrinsically would account for the observed differences. Our dataset was too small to allow for reliable comparison of implant types.

An apparent explanation for the observed difference in revision risk is that the surgeons participating in the trial had received extensive training in using the implant for well-defined indications. Moreover, the trial surgeons also performed the PFA procedure roughly 7 times as often as the nontrial surgeons within this study’s accrual period. Factors such as training and surgeon volume are well-established prognosticators for surgical outcomes, and high surgeon volume is associated with a lower risk of revision in knee arthroplasty.^23,24,25,26^ One may surmise that this is not only reflected in the surgical outcome but extends to surgeons’ patient selection criteria as well as their indication threshold for both the primary procedure and subsequent revisions. Our data were insufficient to assess any association between surgeon training and patient selection. Other mechanisms, such as institutional expectations and personal factors, may also play a role. Thus, the higher number of PFA procedures performed by trial surgeons prompted suspicion that increased experience with PFA surgery may be associated with lower revision rates. Yet adjusting for the PFA experience revealed no clear pattern in revision rates, suggesting the presence of an association with training among the surgeons. It should be noted, however, that limited data were available for this analysis.

Our study presents a novel approach to the debate on whether to recommend one type of knee implant over another (eg, PFA vs TKA), and we have attempted to emphasize the necessity of assessing the reliability of the data that discussions like these are founded on. Within the limitations of this study, this study observed an association between surgeon training and the risk of revision (6 years after the procedure). Factors such as surgeon training and experience are not commonly accounted for in registry-based studies, and we believe that unobserved confounding bias, for instance caused by the level of implant-related surgeon experience, is likely present in many implant registry–based studies. This bias has important consequences when interpreting the results of such studies and the strength of evidence they provide. Registry revision data may be misleading or misinterpreted if confounders, such as the aforementioned, have greater influence on the analysis results than the inherent properties of implants. This confounding may have unjustly led to a suggestion to limit the use of PFA implants.^7^ Furthermore, we believe that this perspective is applicable to other implant types, or even surgical procedures in general, when judging the relative merits of implants from registry data. On an even larger scale, a recent study by Anderson et al^27^ found evidence of potentially important unobserved confounding at the patient-level among National Health Service and private hospitals in England, which corroborates the importance of thorough data collection and assessment, as unobserved confounding may be present at multiple levels.

We want to stress that we find registry data valuable, but that they should be analyzed and interpreted with caution. Aberrant observations, for example, a high implant revision rate, should prompt further investigations to explain these findings. This investigation will most often require detailed and valid information about individual treatment courses that is not readily available in registries. Currently, this requires a much higher effort than performing analyses simply from registries, but we believe that the quality of assessments will be worth the effort.

### Strengths and Limitations

There are strengths to this analysis. First, the use of Danish health registries allowed for complete follow-up of all included patients. The combination of both registry data and individual hospital notes provided a detailed description of each included knee as well as of the sequence of postoperative events. Second, a detailed statistical analysis plan had been prespecified. To our knowledge, it is still not common practice to prespecify such a plan for registry-based studies, although it increases transparency and confidence in observational research.^28,29^ Third, several sensitivity analyses were performed and were consistent with the main findings.

The study also has limitations. First, the study was limited by missing data and possible incompleteness of the registries; and as a result, we may have missed data on confounders. However, we believe that we identified the most important confounders in this analysis through expert discussion. The issue of missing data may have skewed the results such that important clinical variables were overlooked. Second, a major limitation to this study is the modest number of trial surgeons, which may reduce generalizability due to the select group of surgeons, and the limited data available to account for the level of surgeon experience with PFA. Surgeons may have performed PFA prior to the accrual period of this study; however, these data were unavailable. Third, another major limitation was the inability to perform reliable comparison of implant types used on the knees by trial and nontrial surgeons. Additionally, this study mostly focused on the number of revisions without assessing the causes for revision. Some clinical variables may be considered more important than others when evaluating the indications for revision surgery, and surgeons with various levels of experience may perceive the relative importance of these variables differently.

## Conclusions

This cohort study found that the cumulative 6-year revision rate for patients who underwent PFA was lower among knees operated on by knee surgeons who received intensive PFA training prior to trial participation compared with knee surgeons who had not. Our study highlights the association of surgeon training with revision risk of PFA, showing a risk of revision 3 times lower for knees operated on by surgeons trained specifically for the procedure. Registries do not account for the level of surgeon training, and this may be a potential important confounder in registry-based studies. Such confounding may be extrapolated to other types of registry-based analyses and has important consequences when interpreting their results.

## Notes

Supplement 1.
eMethods.
**eTable 1.** Surgical data of all the included knees, stratified by exposure group **eTable 2.** Sensitivity analysis results for the six-year risk ratio of revision, reoperation, and mortality, via propensity score weighting **eFigure 1.** Supplementary analyses for the adjusted six-year revision risk ratio for knees operated by trial-surgeons vs. nontrial surgeons based on multiple logistic regression **eFigure 2.** Further adjusted six-year reoperation risk ratio for trial-surgeons vs. nontrial surgeons based on multiple logistic regression (none), including adjustment for potential confounders (y-axis) **eFigure 3.** Further adjusted six-year mortality risk ratio for trial-surgeons vs. nontrial surgeons based on multiple logistic regression (none), including adjustment for potential confounders (y-axis)

Supplement 2.
Data Sharing Statement
