# Errors in Author Affiliations and Abstract

**DOI:** 10.1001/jamanetworkopen.2025.28720

**Published:** 2025-07-30

In the Original Investigation titled “Surgeon Training and Revision Rates After Patellofemoral Arthroplasty,”^1^ published June 27, 2025, there were errors in the Author Affiliations and the Abstract. An affiliation was missing for Dr Louise E. Rasmussen, and there was a typo in the Abstract. This article has been corrected.^1^
