# Error in Figure 1

**DOI:** 10.1001/jamanetworkopen.2025.41442

**Published:** 2025-10-06

In the Original Investigation titled “Surgeon Training and Revision Rates After Patellofemoral Arthroplasty,”^1^ published June 27, 2025, there was an error in Figure 1. Among 208 knees operated on by nontrial surgeons, the number of deaths should have been listed as 12. This article has been corrected.^1^
