# Supplementary material for: Surgeon Training and Revision Rates After Patellofemoral Arthroplasty
Source: JAMA Netw Open. 2025 Jun 27;8(6):e2517825. doi: 10.1001/jamanetworkopen.2025.17825 (PMC12205405; doi:10.1001/jamanetworkopen.2025.17825)
Supplement: Supplement 1. — eMethods. eTable 1. Surgical data of all the included knees, stratified by exposure group eTable 2. Sensitivity analysis results for the six-year risk ratio of revision, reoperation, and mortality, via propensity score weighting eFigure 1. Supplementary analyses for the adjusted six-year revision risk ratio for knees operated by trial-surgeons vs. nontrial surgeons based on multiple logistic regression eFigure 2. Further adjusted six-year reoperation risk ratio for trial-surgeons vs. nontrial surgeons based on multiple logistic regression (none), including adjustment for potential confounders (y-axis) eFigure 3. Further adjusted six-year mortality risk ratio for trial-surgeons vs. nontrial surgeons based on multiple logistic regression (none), including adjustment for potential confounders (y-axis) [file jamanetwopen-e2517825-s001.pdf]

Supplementary Online Content

Rasmussen LE, Hoffmann AG, Blanche P, et al. Surgeon training and revision rates after patellofemoral arthroplasty. *JAMA Netw Open*. 2025;8(6):e2517825. doi:10.1001/jamanetworkopen.2025.17825

eMethods.
eTable 1. Surgical data of all the included knees, stratified by exposure group
eTable 2. Sensitivity analysis results for the six-year risk ratio of revision, reoperation, and mortality, via propensity score weighting
eFigure 1. Supplementary analyses for the adjusted six-year revision risk ratio for knees operated by trial-surgeons vs. non-trial surgeons based on multiple logistic regression
eFigure 2. Further adjusted six-year reoperation risk ratio for trial-surgeons vs. non-trial surgeons based on multiple logistic regression (none), including adjustment for potential confounders (y-axis)
eFigure 3. Further adjusted six-year mortality risk ratio for trial-surgeons vs. non-trial surgeons based on multiple logistic regression (none), including adjustment for potential confounders (y-axis)

This supplementary material has been provided by the authors to give readers additional information about their work.

eMethods.

We proceeded as follows for the two-step bootstrap resampling approach, in the sensitivity analysis that accounts for a potentially non-negligible correlation between the outcomes of two surgeries performed by the same surgeon.
First, we randomly draw surgeons with replacement among all surgeons (s=1,...,S). Then, for each randomly drawn surgeon 's', we randomly sample 'm_s' patients among his 'n_s' patients, with replacement. To preserve the important feature of the data that the number of patients by surgeon varies, and that it might be informative, we randomly draw a number of patients such that i) the number of patients per surgeon in each bootstrap sample ('m_s') is proportional to that of the actual data ('n_s') and ii) we made sure that all bootstrap samples have approximately the same sample size (M) as the actual data set (n). The latter is just a small sample correction (asymptotically, it becomes negligible). Specifically, let 'N' (resp. 'M') be the sum of all 'n_s' (resp. 'm_s') over all randomly sampled surgeons 's'. We made sure that 'M' (i.e., the total number of patients in the bootstrapped sample) is approximately n (i.e., the sample size of our data set, which is equal to the sum of all 'n_s' over all surgeons s of the original data set) by setting m_s= (n_s/N)*n patients for each surgeon s (note: m_s was rounded to the closest integer)."

© 2025 LE Rasmussen et al. *JAMA Network Open*.

eTable 1. Surgical data of all the included knees, stratified by exposure group

Characteristic	Knees operated by trial-surgeons, n = 274[1]	Knees operated by non-trial surgeons, n = 208[1]
Surgery Year		
2008	10 (3.6%)	32 (15%)
2009	16 (5.8%)	27 (13%)
2010	22 (8.0%)	45 (22%)
2011	21 (7.7%)	27 (13%)
2012	26 (9.5%)	30 (14%)
2013	43 (16%)	16 (7.7%)
2014	52 (19%)	15 (7.2%)
2015	84 (31%)	16 (7.7%)
Duration of Surgical Procedure		
Minutes	63 (24)	64 (17)
Not recorded	61	125
Perioperative Pathology PF-OA		
Yes	255 (93%)	196 (94%)
No or not recorded	19 (6.9%)	12 (5.8%)
Perioperative Pathology TF-OA		
Yes	0 (0%)	2 (1.0%)
No or not recorded	274 (100%)	206 (99%)
Implant Brand		
Avon	263 (96%)	47 (23%)
Cartier	0 (0%)	13 (6.5%)
Hemicap	0 (0%)	8 (4.0%)
Journey	3 (1.1%)	36 (18%)
LCS	0 (0%)	17 (8.5%)
Sigma	0 (0%)	18 (9.0%)
Vanguard	7 (2.6%)	19 (9.5%)
Wave	0 (0%)	24 (12%)
Zimmer	1 (0.4%)	19 (9.5%)
Not recorded	0	7

[1] n (%); Mean (SD)

PF-OA, patellofemoral osteoarthritis; TF-OA, tibiofemoral osteoarthritis

© 2025 LE Rasmussen et al. *JAMA Network Open*.

eTable 2. Sensitivity analysis results for the six-year risk ratio of revision, reoperation, and mortality, via propensity score weighting

	**Knees operated by trial-surgeons, n = 274[1]**	**Knees operated by non-trial surgeons, n = 208[1]**	**Risk Ratio (propensity score weighting)**
Revision	22 (8.0%)	54 (26%)	0.35 [0.21, 0.61]
Reoperation	33 (12%)	40 (19%)	0.75 [0.45, 1.25]
Mortality	18 (6.6%)	12 (5.8%)	1.21 [0.53, 2.80]

[1] n (%); Mean (SD)

© 2025 LE Rasmussen et al. *JAMA Network Open*.

eFigure 1. Supplementary analyses for the adjusted six-year revision risk ratio for knees operated by trial-surgeons vs. non-trial surgeons based on multiple logistic regression. The analysis using the main adjustment set as well as those additionally adjusting on either surgeon experience, BMI, tibiofemoral Kellgren-Lawrence (TF-KL) grade, and patellofemoral Kellgren-Lawrence (PF-KL) grade are presented. For completeness, the sample sizes of the two groups for each of these analyses are provided. Because of missing data and because complete case analyses were performed, the sample sizes differ.

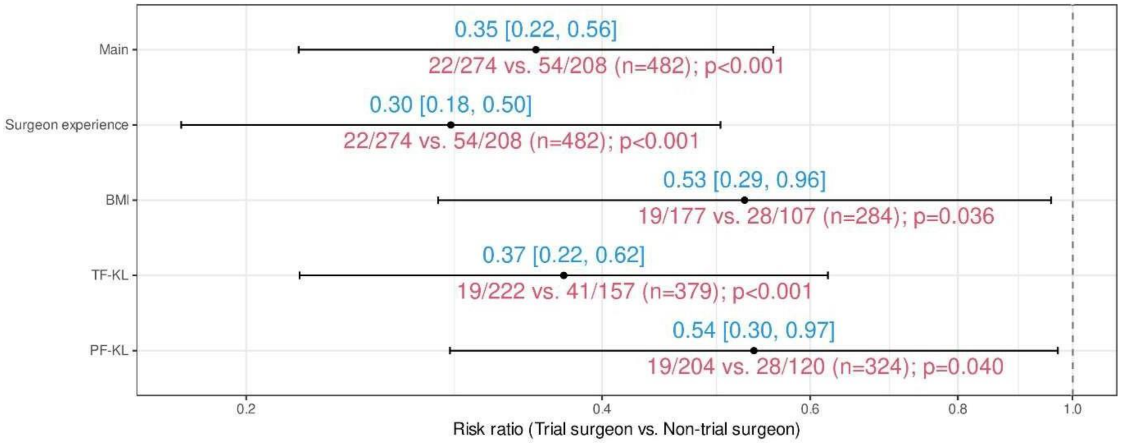

© 2025 LE Rasmussen et al. *JAMA Network Open*.

eFigure 2. Further adjusted six-year reoperation risk ratio for trial-surgeons vs. non-trial surgeons based on multiple logistic regression (none), including adjustment for potential confounders (y-axis)

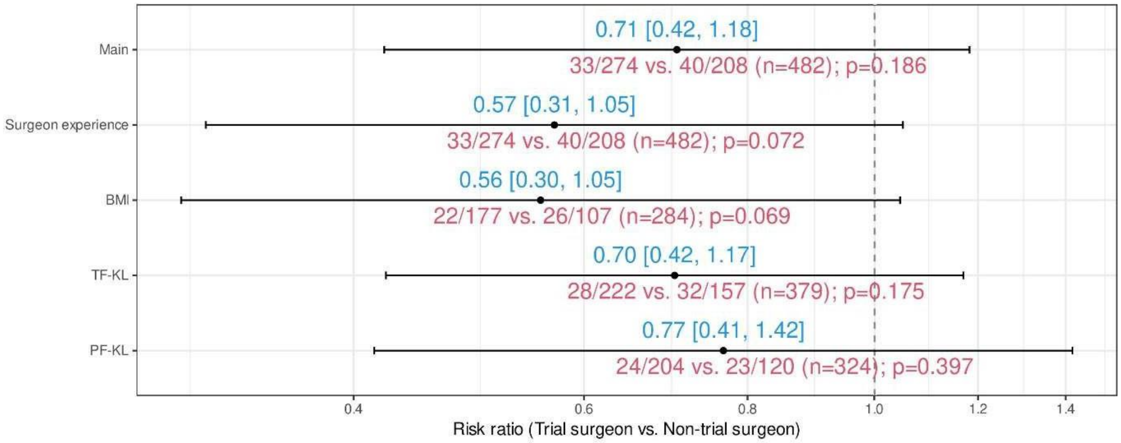

© 2025 LE Rasmussen et al. *JAMA Network Open*.

eFigure 3: Further adjusted six-year mortality risk ratio for trial-surgeons vs. non-trial surgeons based on multiple logistic regression (none), including adjustment for potential confounders (y-axis).

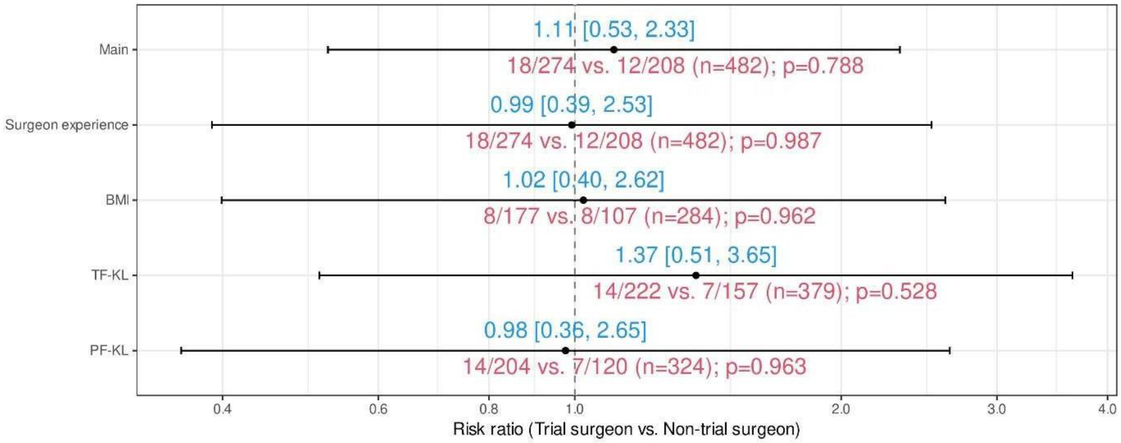

© 2025 LE Rasmussen et al. *JAMA Network Open*.
