# Supplementary material for: Surgeon Training and Revision Rates After Patellofemoral Arthroplasty
Source: JAMA Netw Open. 2025 Jun 27;8(6):e2517825. doi: 10.1001/jamanetworkopen.2025.17825 (PMC12205405; doi:10.1001/jamanetworkopen.2025.17825)
Supplement: Supplement 2. — Data Sharing Statement [file jamanetwopen-e2517825-s002.pdf]

Data Sharing Statement

Rasmussen. Surgeon Training and Revision Rates After Patellofemoral Arthroplasty. *JAMA Netw Open*. Published June 27, 2025. doi:10.1001/jamanetworkopen.2025.17825

Data
Data available: No

Additional Information
Explanation for why data not available: The data set and the hospital notes that support the findings of this study are available through the Danish Health Data Authority's Research Services and the National Board of Health in the Capital Region of Denmark upon access request. Thus, the data are not publicly available, and access must be obtained with permission from both institutions as well as through a Danish research institution.
